# HIV Serostatus Disclosure and Its Predictors Among Children Living With HIV in Ethiopia: A Systematic Review and Meta-Analysis

**DOI:** 10.3389/fpubh.2022.859469

**Published:** 2022-06-02

**Authors:** Tesfanesh Lemma, Mulualem Silesh, Birhan Tsegaw Taye, Kelem Desta, Tebabere Moltot Kitaw, Tiwabwork Tekalign

**Affiliations:** ^1^School of Nursing and Midwifery, Asrat Woldeyes Health Science Campus, Debre Berhan University, Debre Berhan, Ethiopia; ^2^School of Nursing, Arba Minch University College of Medicine and Health Science, Arba Minch, Ethiopia

**Keywords:** children, disclosure, HIV, serostatus disclosure, Ethiopia

## Abstract

**Background:**

HIV disclosure among children refers to when the caregiver is having disclosed to the child that he or she has HIV specifically. Disclosure significantly improved adherence to treatment and quality of life among children living with HIV/AIDS. Even though, the benefits of disclosure are considerable, informing a child of his or her own HIV status is often delayed. There is a dearth of studies on HIV serostatus disclosure among children in Ethiopia. Therefore, this study aimed to assess the pooled prevalence of HIV serostatus disclosure and associated factors among children living with HIV in Ethiopia.

**Methods and Materials:**

Using a combination of search terms and Boolean operators, studies were retrieved from Pub Med/MEDLINE, EMBASE, CINAHL, Science Direct, Scopus, Web of Science, Cochran library, and Google Scholar. Five authors independently assessed the quality of each study using the modified Newcastle Ottawa Scale (NOS) for cross-sectional studies. STATA Version 11 software was used for statistical analyses. The random-effects (Der Simonian and Laird) method was used for the meta-analysis. The heterogeneity test was carried out with the help of *I*-squared (*I*^2^) statistics. A leave-one-out sensitivity analysis was carried out.

**Results:**

A total of 12 articles with 3,410 participants were included in this systematic review and meta-analysis. The pooled prevalence of HIV serostatus disclosure among children was 36.87% (95% CI: 29.30, 44.44; *I*^2^ = 95.8%). Children aged older than 10 years (*p* = 0.003) and caregivers with primary and above education (*p* < 0.001) were factors significantly associated with HIV serostatus disclosure among children.

**Conclusions:**

The finding of this study showed that HIV serostatus disclosure among children is relatively low. Therefore, developing clear guideline on HIV serostatus disclosure among children, strengthening public health education or community awareness creation about HIV/AIDS to promote the benefits of disclosure and extensively provision of counseling by health care providers are essential to enhance HIV serostatus disclosure among children.

**Systematic Review Registration:**

https://www.crd.york.ac.uk/prospero/display_record.php?ID=CRD42021239035.

## Introduction

Human immunodeficiency virus infection/Acquired immune deficiency Syndrome (HIV/AIDS) is still a major public health problem around the world (1). Globally, in 2020, there were 37.7 million (30.2−45.1 million) people living with HIV (PLWH) and 1.7 million children (0–14 years) have been infected with HIV and 99,000 children have died of HIV (2, 3); about 20.7 million PLWH live in Eastern and Southern Africa (4). Besides, Ethiopia has achieved great progress in combating the HIV/AIDS epidemic over the last decade, HIV was still one of public health problem in the country; the prevalence remains extremely high in urban areas, with estimates indicating a 3% prevalence rate compared to <1% nationally (5). In Ethiopia nearly 620,000 adults and children were living with HIV; of which 44,000 were children <15 years old in 2020. In addition, 13,000 of adults and children died due to AIDS (6).

HIV disclosure among HIV-infected children refers to when the caregiver has disclosed to the child that he or she has HIV infected (7, 8). Disclosing one's HIV-positive serostatus can help both HIV-positive people and public health preventive initiatives (9). In HIV-positive children, disclosure enhanced clinical outcomes such as viral suppression, drug adherence, retention in care, and HIV knowledge (10–12). However, one of the most difficult decisions for parents is whether or not to tell their children about their HIV status (9).

According to World Health Organization (WHO) guidelines on HIV counseling for children under the age of 12, children of school age should be informed of their HIV-positive status (13). HIV status disclosure significantly improved adherence to treatment and quality of life among children living with HIV/AIDS (14). Furthermore, disclosing HIV-positive serostatus has advantages for children, parents, and family dynamics (15). However, disclosing a child's HIV status has its own set of difficulties (16). Disclosure requires providing persons with access to appropriate physical and psychological support, which is a critical component of secondary prevention (17). Many people have their own particular disclosure strategies and objectives, which are carefully considered to achieve beneficial results (18).

Treatment must be scaled up even more, especially for HIV-infected children. Globally, only 54% (37%−69%) of children (0–14 years old) were receiving ART at the end of 2020 (3). Also, only 38% of them were on antiretroviral therapy (ART) in Ethiopia (19). HIV disclosure can improve social support, antiretroviral adherence, and healthcare attendance for people living with HIV (20, 21). Despite this, children's HIV disclosure remained low. According to one systematic review, in low- and middle-income countries the overall rate of HIV-positive status disclosure to HIV-infected children was low, which ranges from 1.7 to 41% (7).

Furthermore, disclosure of HIV status among children living with HIV has both beneficial and challenging aspects (7). Developing countries have a lower rate of HIV-positive status disclosure than developed countries (22). Even though, the benefits of disclosure are considerable, informing a child of his or her own HIV status is often delayed (23). This systematic review and meta-analysis sought to fill the gaps in the Ethiopian literature by estimating the pooled effect of disclosure status among children living with HIV in Ethiopia. We believe that our findings will be relevant to those involved in designing interventions to improve HIV serostatus disclosure among HIV-infected children.

### Objectives of the Review

#### General Objectives

- To assess the HIV serostatus disclosure and its predictors among children living with HIV in Ethiopia.

#### Specific Objectives

- To determine the pooled prevalence of HIV serostatus disclosure among children in Ethiopia.
- To identify the predictors of HIV serostatus disclosure among children in Ethiopia.

## Methods and Materials

### Study Design and Search Strategy

The protocol was registered with in PROSPERO (https://www.crd.york.ac.uk/), registration number (ID: CRD42021239035). This systematic review and meta-analysis were conducted under the guidelines of the Preferred Reporting Items for Systematic Reviews and Meta-analyses (PRISMA) statement (24, 25). All published articles were searched from major international databases like PubMed /MEDLINE, EMBASE, CINAHL, Science Direct, Scopus, Web of Science, Cochran library, and Google Scholar. A second search was done by using all identified keywords and index terms across all included databases. Also, the reference list of all identified reports and articles was searched for additional studies. The search was performed using key terms such as HIV Disclosure, Disclosure of HIV, HIV positive status disclosure, HIV status disclosure, Disclosure of HIV status, HIV related disclosure, Disclosure of HIV serostatus, Children, Young children's, pediatrics, childhood, children's, pediatrics, pediatric and Ethiopia. The searching periods were from October 1 to 31, 2021.

### Study Selection and Eligibility Criteria

Study participants included HIV positive children who were booked in ART clinic in Ethiopia. This study included all published and unpublished observational (cross-sectional and case–control) studies on the HIV serostatus disclosure among children in Ethiopia. This review included studies done until November 1, 2021, and was written in the English language.

### Data Extraction and Quality Appraisal

The data were extracted by three independent authors (TL, MS, and BT) using a data extraction format prepared in a Microsoft Excel 2010 spread sheet. The extracted data were: the first author's name, publication year, study area, region, study design, sample size, sampling method, HIV serostatus disclosure, and associated factors with their odds ratio. The quality of each study was assessed using the modified Newcastle-Ottawa Scale (NOS) for cross-sectional studies (26). The tool's key components were methodological quality, comparability, outcomes, and statistical analysis of each original study, which were graded from five to two and three stars, respectively. The NOS included three categorical criteria with a maximum score of 10 points. Studies with a medium score (50% of quality evaluation criteria) and above were included in the analysis (27). The quality of each study was evaluated independently by five authors (TL, MS, TT, KD, and TM) and any disagreements were resolved by discussion and consensus.

### Publication Bias and Heterogeneity

To assess the existence of publication bias, funnel plots were used and Egger's test was computed. A *p*-value < 0.05 was used to declare the statistical significance of publication bias. *I*^2^ test statistics were used to check the heterogeneity of studies. *I*^2^ test statistics of < 50, 50–75, and > 75% were declared as low, moderate, and high heterogeneity respectively (28).

### Outcome

HIV serostatus disclosure.

### Exposure

Determinants of HIV serostatus disclosure. The only factor identified as a significant factor in the two and above studies was included in this review and meta-analysis.

### Data Synthesis and Analysis

STATA version11 software was used to conduct the analysis. The heterogeneity test was conducted by using *I*-squared (*I*^2^) statistics. The pooled prevalence of HIV serostatus disclosure was carried out using a random-effects (Der Simonian and Laird) method. To minimize the potential random variations between studies; the sources of heterogeneity were analyzed using subgroup analysis, and meta-regression. A sensitivity analysis was also conducted.

## Results

### Study Screening

**Figure 1:**
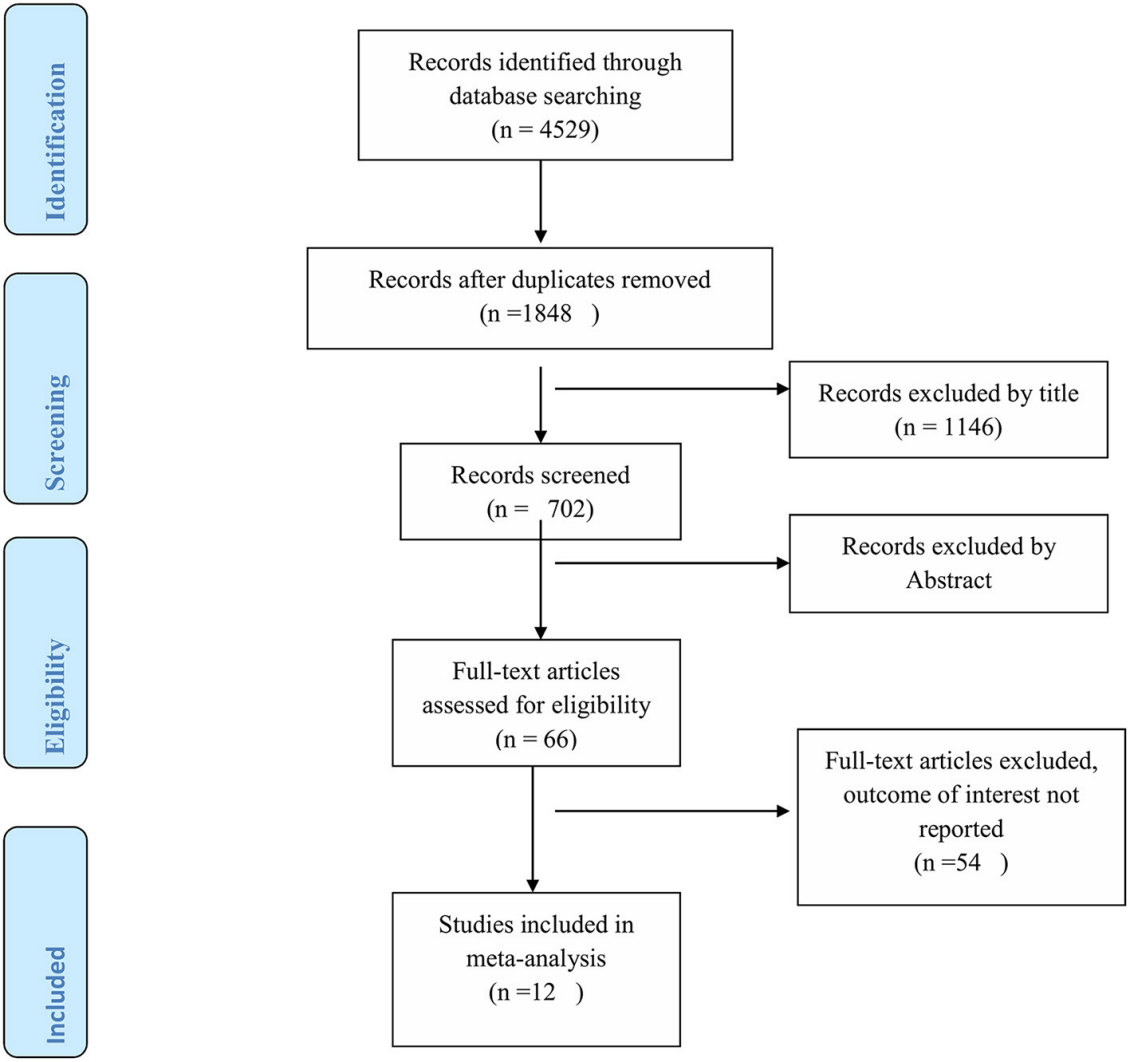
PRISMA flow diagram of study selection.

Initially, a total of 4,529 studies were retrieved from the databases and manual searching. From this, 1,848 duplicates were found and removed. Their title screened the remaining 1,146 articles, and abstract 636 irrelevant studies were removed. Sixty-six full-text articles were assessed for eligibility, and 52 of them were excluded due to not reporting the outcome of interest, and two articles with poor methodological quality. Finally, a total of 12 studies fulfilled the inclusion criteria and enrolled in the study (Figure 1).

### Study Characteristics

A total of 12 (29–40) articles with 3,410 participants were included in this systematic review and meta-analysis. All the included studies were cross-sectional studies and the sample size ranged from 172 (33) to 428 (39). Most studies were conducted in the Amhara region. Among the included studies, HIV serostatus disclosure among children ranged from 16.3 (33) to 60.6 (35).

### HIV Serostatus Disclosure Among Children

**Figure 2:**
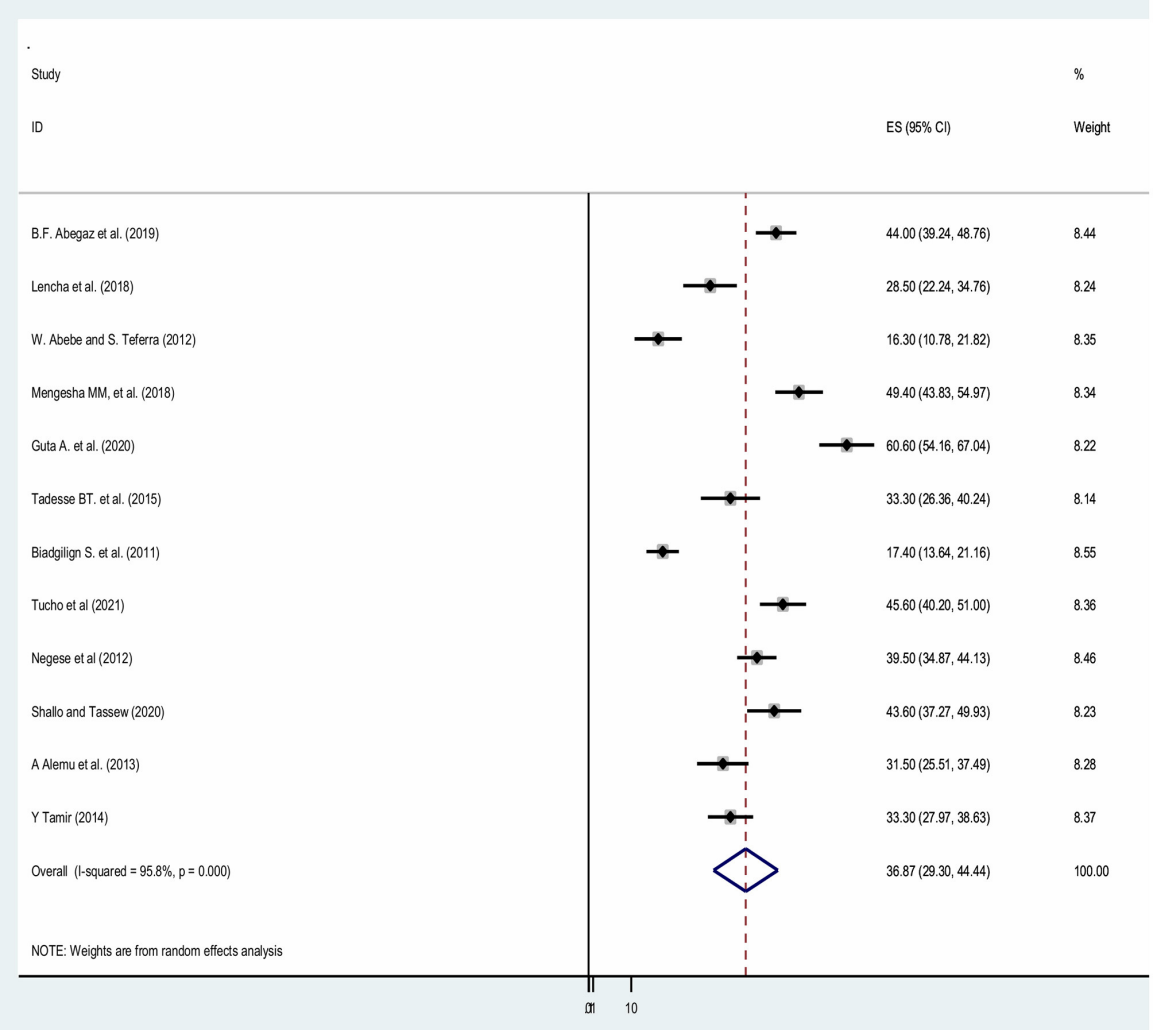
Forest plot showing the pooled prevalence of HIV serostatus disclosure among children in Ethiopia.

By including the 12 published research articles we had estimated the pooled prevalence of HIV serostatus disclosure among children in Ethiopia. Accordingly, the overall estimated pooled prevalence of HIV serostatus disclosure among children with a random-effects model was 36.87% (95% CI: 29.3, 44.4) with a heterogeneity index (*I*^2^) of 95.8% (*p* < 0.001; Figure 2).

### Subgroup Analysis

**Figure 3:**
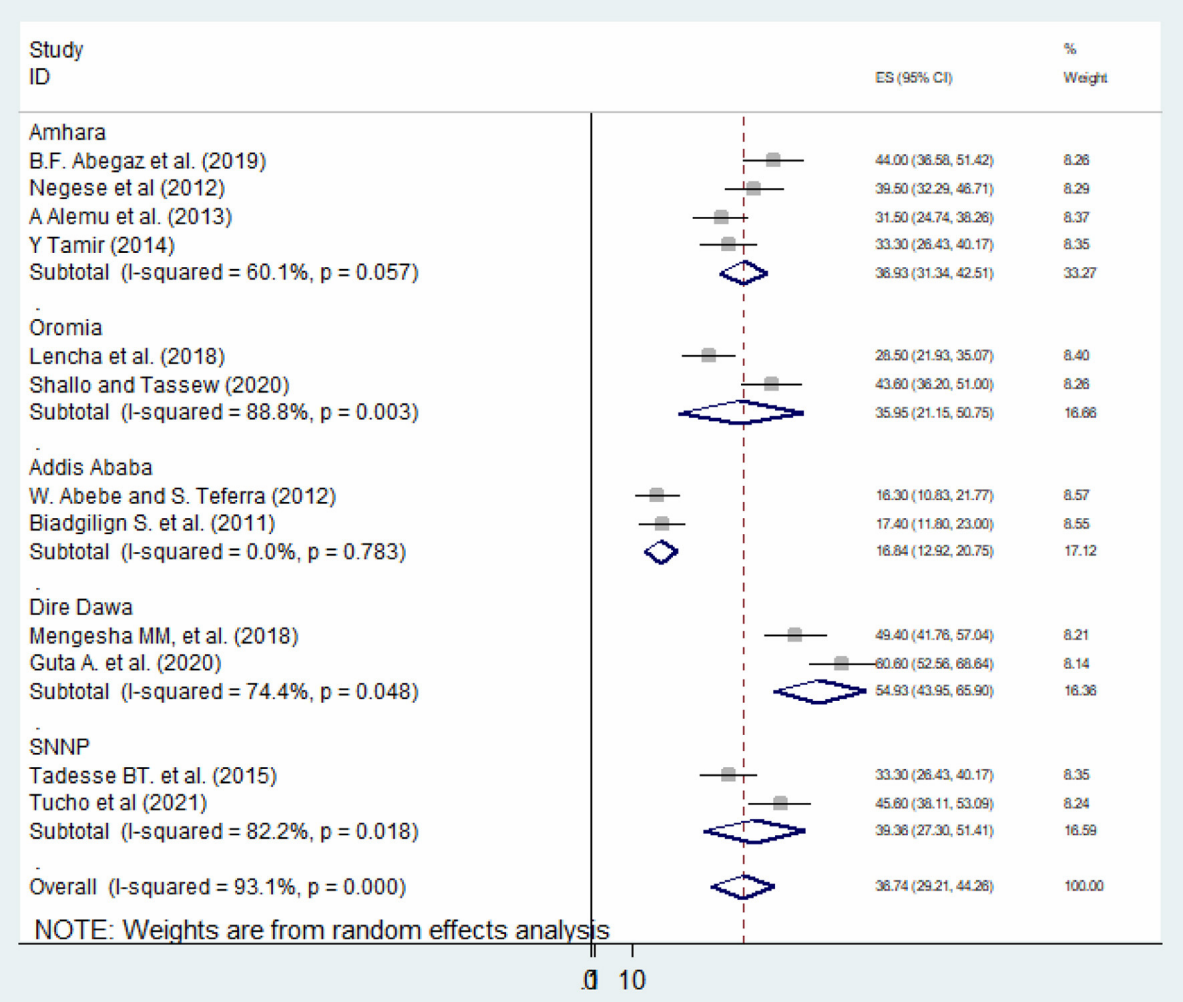
Subgroup analysis of the prevalence of HIV serostatus disclosure among children in Ethiopia.

Subgroup analyses revealed a marked variation across regions. Based on the subgroup analysis result, the highest (54.92%; 95% CI: 43.95, 65.90), *I*^2^ = 74.4%) seen in Dire Dawa region and the lowest (16.83%; 95% CI: 12.92, 20.75), *I*^2^ = 0%) seen in Addis Ababa (Figure 3).

### Heterogeneity and Publication Bias

**Figure 4:**
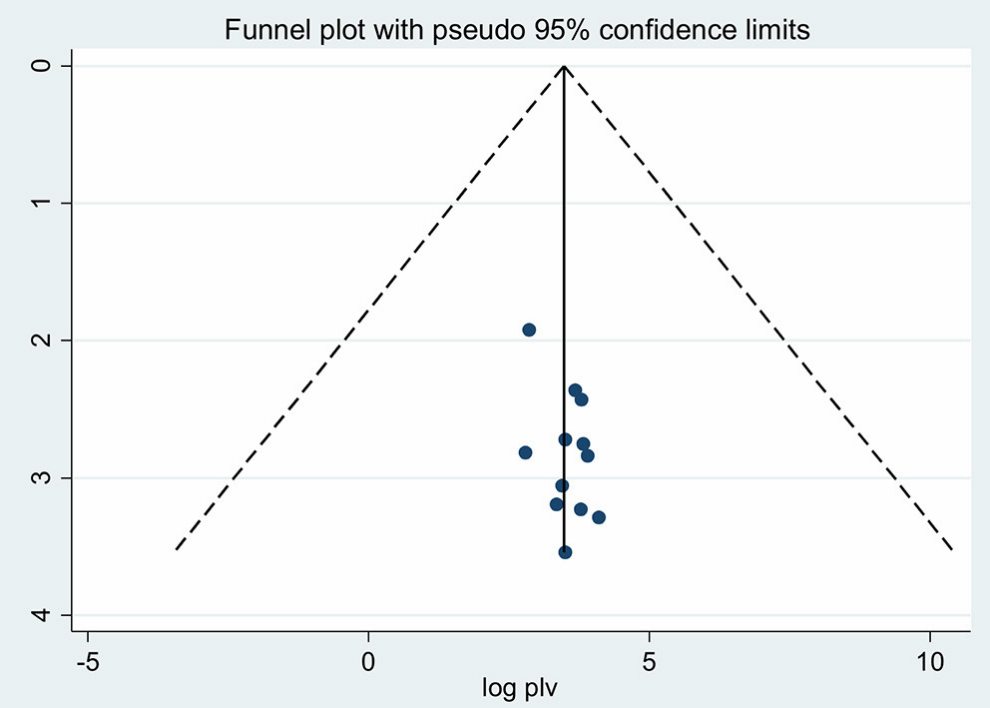
Funnel plot to test the publication bias in 12 studies with 95% confidence limits.

Meta-regression was conducted to identify the source of heterogeneity using sample size and publication year as a covariates. It was indicated that there is no effect of sample size on heterogeneity between studies. But, the year of publication showed that there is heterogeneity between the studies (Table 1). The presence of publication bias was checked using the Egger's test, and a graphical Funnel plot, the result of egger's test was found significant (*p* < 0.001), as a result of estimating the number of missing studies that might exist in a meta-analysis we conducted Duval and Tweedie's trim and fill analysis, but is not significant. Also, visual inspection of the funnel plot indicated asymmetrical distribution showing publication bias (Figure 4).

**Table 1:** Meta-regression analysis of factors affecting between-study heterogeneity.

**Heterogeneity source**	**Coefficients**	**SE**	***p*-value**
Sample size	0.0187308	0.0439353	0.679
Publication year	2.693511	0.7247188	0.004

### Sensitivity Analysis

**Figure 5:**
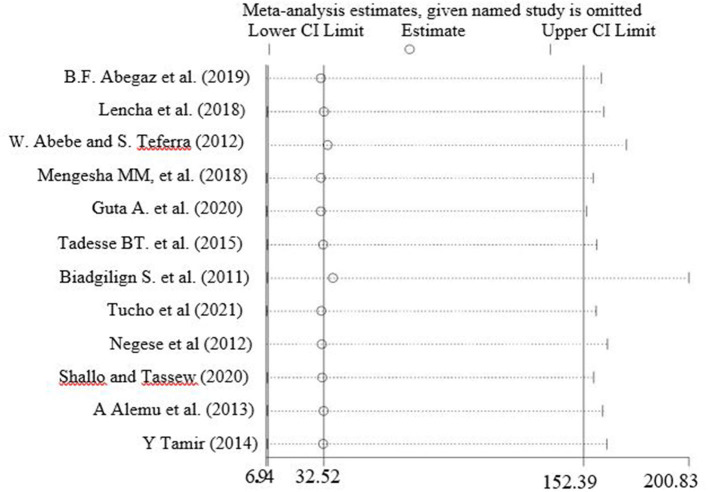
Sensitivity analysis of pooled prevalence for each study being removed one at a time.

All studies have been included in the review, then a sensitivity analysis performed by study quality by leave-one-out studies step by step to evaluate the effect of a single study on the overall effect estimate. The result indicated removing a single study did not have a significant influence on pooled prevalence (Figure 5).

### Factors Associated With HIV Serostatus Disclosure Among Children

Six variables were extracted to identify factors associated with HIV serostatus disclosure among children. Of these, age of children and educational status of caregivers were found to be significantly associated with HIV serostatus disclosure among children (Table 2).

**Table 2:** Factors associated with HIV serostatus disclosure among children in Ethiopia.

**Determinants**	**Comparisons**	**Number of studies**	**Sample size**	**OR (95% CI)**	***p*-value**	***I*^2^ (%)**	**Heterogeneity test (*p*-value)**
Age	<10 vs. ≥10 years	9	2,600	11.15 (4.82–11.98)	0.003	66.2	<0.001
Duration of ART	≤ 5 vs. >5 years	4	979	6.67 (4.18–10.65)	0.176	39.2	<0.001
Care givers education	No formal vs. primary and above	4	1,340	3.21 (1.01–10.18)	<0.001	93.8	0.047
Death of family	Yes vs. no	2	649	2.49 (1.76–3.51)	0.301	6.5	<0.001
Sex	Male vs. female	2	457	2.4 (1.63–3.53)	0.862	0	<0.001
Age at diagnosis of HIV	≤ 5 vs. >5 years	2	431	3.31 (2.16–5.07)	0.774	0	<0.001

Children whose aged older than 10 years were 11 times more likely to know their HIV positive status compared to their counterparts (AOR = 11.15, 95% CI: 4.82–11.98), *p* = 0.003, *I*^2^: 66.2%, the heterogeneity test (*p* < 0.001). Those caregivers of HIV-positive children with primary and above education were three times more likely to have disclosed HIV status to their children compared to those with no formal education (AOR = 3.21, 95% CI: 1.01–10.18), *p* = <0.001, *I*^2^: 93.8%, the heterogeneity test (*p* < 0.001).

## Discussion

In this systematic review and meta-analysis, the overall pooled prevalence of HIV serostatus disclosure among children was 36.87% (95% CI: 29.30, 44.44). This result is relatively low, because according to WHO guidelines children of school age should be informed of their HIV-positive status (13). According to the results of the subgroup analysis, the highest prevalence of HIV serostatus disclosure among children was found in the Dire Dawa region (54.92%), while the lowest prevalence of disclosure rate was found in Addis Ababa (16.83%). The possible variation might be due to the difference in study period; because community awareness about HIV and related information improve over time which might increase the disclosure by the caregivers. Also, socio-cultural difference might have role for the difference between these studies.

In the current study, child age was significantly associated with HIV serostatus disclosure. Children whose aged older than 10 years were 11 times more likely to know their HIV positive status compared to their counterparts (AOR = 11.15, 95% CI: 4.82–11.98), *p* = 0.003, *I*^2^: 66.2%, the heterogeneity test (*p* < 0.001). This is consistent with a review conducted in Sub-Saharan Africa (7, 41). This might be because the caregivers may have believed that the older children were mature enough to understand the illness and recognize the complex causes and consequences of the illness. Also, children in these age groups were more interested in knowing why they were taking medication and what their illness was like.

Furthermore, parental/care giver's educational status also significantly associated with HIV serostatus disclosure among children. Those caregivers of HIV-positive children with primary and above education were three times more likely disclosed the HIV status to their children compared to those care givers with no formal education (AOR = 3.21, 95% CI: 1.01, 10.18), *p* = <0.001, *I*^2^: 93.8%, the heterogeneity test (*p* < 0.001). This is consistent with the review conducted in resource-limited settings (42). This could be because caregivers with a higher educational level have been exposed to more information about disclosure which allowing them to understand the importance of disclosure and well prepared to handle the disclosure process.

This review showed that HIV serostatus disclosure among children in Ethiopia was not sufficient enough which is difficult to achieve the global target of HIV free generation and sustainable development goal 3 which aims to ensure healthy lives and promote wellbeing for all at all ages, including people living with HIV. Therefore, this review informed the following recommendations; first, healthcare providers should give more attention on the purpose of disclosure to infected children. Furthermore, public health education/ community education should be strengthen to promote HIV/AIDS as a shared burden which eventually enhance community/familial acceptance of living with HIV infected children. Finally, further studies are needed to identify other determinants of HIV serostatus disclosure practices among children in resource-limited countries.

### Strength and Limitation of the Study

The strength of this review were the study was conducted using a very thorough systematic search, with an international standardized protocol for the search strategy and internationally approved tools for a critical appraisal system for each study's quality assessment. However, only three regions and two administrative cities out of nine regional states were included in this analysis that might reduce its representativeness for the country. Also, lack of studies.

## Conclusions

According to the findings of this study, only one-third of caregivers disclosed their child's HIV serostatus. Age of infected children and the educational status of care givers were found to be significantly associated with HIV serostatus disclosure among children. As a result, authors suggest that designing strategies by developing clear guideline on HIV serostatus disclosure among children, strengthening parental/caregiver health education/training or community awareness creation about HIV disclosure and HIV/AIDS related information to promote the benefits of disclosure and extensively provision of counseling by health care providers are essential to enhance HIV serostatus disclosure among children.

## Data Availability Statement

The datasets presented in this study can be found in online repositories. The names of the repository/repositories and accession number(s) can be found in the article/Supplementary Material.

## Author Contributions

All authors made a significant contribution to the work reported, whether that is in the conception, study design, execution, acquisition of data, analysis, and interpretation, or in all these areas, took part in drafting, revising, or critically reviewing the article, gave final approval of the version to be published, have agreed on the journal to which the article has been submitted, and agree to be accountable for all aspects of the work.

## Conflict of Interest

The authors declare that the research was conducted in the absence of any commercial or financial relationships that could be construed as a potential conflict of interest.

## Publisher's Note

All claims expressed in this article are solely those of the authors and do not necessarily represent those of their affiliated organizations, or those of the publisher, the editors and the reviewers. Any product that may be evaluated in this article, or claim that may be made by its manufacturer, is not guaranteed or endorsed by the publisher.

## References

[1] Osingada CP Okuga M Nabirye RC Sewankambo NK Nakanjako D. Disclosure of parental HIV status to children : experiences of adults receiving antiretroviral treatment at an Urban Clinic in Kampala, Uganda. AIDS Res Treat. (2017) 2017:3458684. 10.1155/2017/3458684 29209538 PMC5676343

[2] UNAIDS. Global HIV statistics. FACT SHEET – WORLD AIDS DAY 2021. Geneva (2021), p. 1–6.

[3] World Health Organization (WHO). HIV_AIDS. Geneva: WHO (2021).

[4] UNAIDS. Global HIV & AIDS statistics - 2020 fact sheet. Ending the Aids Epidermics. Luxembourg (2020), p. 8.

[5] USAIDS. USAID Ethiopia Fact Sheet - HIV and AIDS. Addis Ababa (2020), p. 1–2.

[6] UNAIDS. HIV and AIDS Estimates Adults and children living with Country factsheets ETHIOPIA | 2020 HIV. New York (2020), p. 1–6.

[7] Britto C Mehta K Thomas R Shet A. Prevalence and correlates of HIV disclosure among children and adolescents in low- and middle-income countries: a systematic review. J Dev Behav Pediatr. (2016) 37:496–505. 10.1097/DBP.0000000000000303 27262128 PMC5949066

[8] Wiener L. Disclosure of an HIV diagnosis to children: history, current research, and future directions. J Dev Behav Pediatr. (2008) 28:155–66. 10.1097/01.DBP.0000267570.87564.cd 17435473 PMC2440688

[9] Conserve DF Hill C Groves AK Maman S Hill C. Effectiveness of interventions promoting HIV serostatus disclosure to sexual partners: a systematic review. AIDS Behav. (2016) 19:1763–72. 25645328 10.1007/s10461-015-1006-1 PMC5101233

[10] Adefalu MO Florence M Issa BA Adefalu AA. Does disclosure of HIV/AIDS status to children with HIV/AIDS affect their does disclosure of HIV/AIDS status to children with HIV/AIDS affect their mental health? J Psychiatry. (2016) 20:1. 10.4172/2378-5756.100039930808419

[11] Ozoya OO. Perspectives and practice of hiv disclosure to children and adolescents by health-care providers and caregivers in sub-Saharan Africa : a systematic review. Front Public Health. (2016) 4:166. 10.3389/fpubh.2016.00166 27570762 PMC4981616

[12] Vreeman RC Scanlon ML Marete I Mwangi A Inui TS. Characteristics of HIV-infected adolescents enrolled in a disclosure intervention trial in western Kenya. AIDS Care. (2015) 27(sup1):6–17. 10.1080/09540121.2015.1026307 26616121 PMC4685612

[13] WHO. Guideline on HIV Disclosure Counselling For Children Up to 12 years of Age. Geneva: WHO (2011). 26158185

[14] Bulali RE Kibusi SM Mpondo BCT. Factors associated with HIV status disclosure and its effect on treatment adherence and quality of life among children 6 – 17 Years on antiretroviral therapy in Southern Highlands Zone, Tanzania : unmatched Case Control Study. Int J Pediatr. (2018) 2018:8058291. 10.1155/2018/8058291 30046314 PMC6038591

[15] Tenzek KE Herrman AR May AR Feiner B Allen M. Examining the impact of parental disclosure of HIV on children: a meta-analysis. West J Commun. (2013) 77:323–39. 10.1080/10570314.2012.719092

[16] Beck-sague CM. Disclosure of their HIV status to infected children : a review of the literature. J Trop Pediatr. (2013) 59:84–9. 10.1093/tropej/fms052 23070738 PMC3693505

[17] Conserve DF Groves AK Maman S. Effectiveness of interventions promoting HIV serostatus disclosure to sexual partners: a systematic review. AIDS Behav. (2015) 19:1763–72. 10.1007/s10461-015-1006-1 25645328 PMC5101233

[18] Take K Messages H. Disclosure of HIV-positive status : towards the development of guidelines, strategies and interventions. The issue and why it ' s important. Toronto (2014).

[19] USAIDS. PEPFAR 2021 Country and Regional Operational Plan (COP/RO) Guidance for all PEPFAR Countries. Washington, DC (2021).

[20] Evangeli M Wroe AL. HIV disclosure anxiety: a systematic review and theoretical synthesis. AIDS Behav. (2017) 21:1–11. 10.1007/s10461-016-1453-3 27406227 PMC5216111

[21] Nichols J Steinmetz A Paintsil E. Impact of HIV-status disclosure on adherence to antiretroviral therapy among HIV-infected children in resource-limited settings : a systematic review. AIDS Behav. (2017) 21:59–69. 10.1007/s10461-016-1481-z 27395433

[22] Berhe TM Lemma L Alemayehu A Ajema D Glagn M Dessu S. HIV-positive status disclosure and associated factors among HIV-positive adult patients attending art clinics at public health facilities of Butajira Town, Southern Ethiopia. AIDS Res Treat. (2020) 2020:7165423. 10.1155/2020/7165423 33204528 PMC7665937

[23] Madiba S Diko C. The consequences of delaying telling children with perinatal hiv about their diagnosis as perceived by healthcare workers in the eastern cape; a qualitative study. Children. (2020) 7:289. 10.3390/children7120289 33322497 PMC7763355

[24] Moher D Liberati A Tetzlaff J Altman DG. Preferred reporting items for systematic reviews and meta-analyses: the PRISMA statement. BMJ. (2009) 339:332–6. 10.1136/bmj.b2535 21603045 PMC3090117

[25] Liberati A Altman DG Tetzlaff J Mulrow C Gøtzsche PC Ioannidis JPA . The PRISMA statement for reporting systematic reviews and meta-analyses of studies that evaluate health care interventions: explanation and elaboration. J Clin Epidemiol. (2009) 62:1–34. 10.1016/j.jclinepi.2009.06.006 19631507

[26] Modesti PA Reboldi G Cappuccio FP Agyemang C Remuzzi G Rapi S . Cross sectional study Newcastle - Ottawa Quality Assessment Scale. PLoS ONE. (2016) 11:1–2. 10.1371/journal.pone.0147601 26808317 PMC4725677

[27] Kassa BG Ayele AD Belay HG Tefera AG Tiruneh GA Ayenew NT . Postpartum intrauterine contraceptive device use and its associated factors in Ethiopia: systematic review and meta-analysis. Reprod Health. (2021) 18:1–12. 10.1186/s12978-021-01273-x 34774058 PMC8590214

[28] Higgins JPT Thompson SG Deeks JJ Altman DG. Measuring inconsistency in meta-analyses. Br Med J. (2003) 327:557–60. 10.1136/bmj.327.7414.557 12958120 PMC192859

[29] Abegaz BF Walle TA Tilahun AD HIV. positive status disclosure and associated factor among HIV infected children in pediatric ART clinics in Gondar town public health facilities, North West Ethiopia, 2018. J Infect Public Health. (2019) 12:873–7. 10.1016/j.jiph.2019.05.018 31213410

[30] Lencha B Ameya G Minda Z Lamessa F Darega J. Human immunodeficiency virus infection disclosure status to infected school aged children and associated factors in bale zone, Southeast Ethiopia: cross sectional study 11 Medical and Health Sciences 1117 Public Health and Health Services. BMC Pediatr. (2018) 18:1–8. 10.1186/s12887-018-1336-z 30442118 PMC6236985

[31] Alemu A Berhanu B Emishaw S. Challenges of caregivers to disclose their children's HIV positive status receiving highly active anti retroviral therapy at pediatric anti retroviral therapy clinics in Bahir Dar, North West Ethiopia. J AIDS Clin Res. (2013) 4:11. 10.4172/2155-6113.1000253

[32] Tamir Y. Disclosure status and associated factors open access among children living with HIV in East Gojjam, Northwest of Ethiopia 2014. Qual Prim. Care. (2015) 23:223–30.

[33] Abebe W Teferra S. Disclosure of diagnosis by parents and caregivers to children infected with HIV: prevalence associated factors and perceived barriers in Addis Ababa, Ethiopia. AIDS Care. (2012) 24:1097–102. 10.1080/09540121.2012.656565 22316133

[34] Mengesha MM Dessie Y Roba AA. Perinatally acquired HIV-positive status disclosure and associated factors in Dire Dawa and Harar, Eastern Ethiopia: a health facility-based cross-sectional study. BMJ Open. (2018) 8:e019554 10.1136/bmjopen-2017-019554 30166287 PMC6119410

[35] Guta A Areri HA Anteab K Abera L Umer A. HIV-positive status disclosure and associated factors among children in public health facilities in Dire Dawa, Eastern Ethiopia: a cross-sectional study. PLoS ONE. (2020) 15:1–12. 10.1371/journal.pone.0239767 33044968 PMC7549787

[36] Tadesse BT Foster BA Berhan Y Tang JW. Cross sectional characterization of factors associated with pediatric HIV status disclosure in southern Ethiopia. PLoS ONE. (2015) 10:1–9. 10.1371/journal.pone.0132691 26167687 PMC4500496

[37] Biadgilign S Deribew A Amberbir A Escudero HR Deribe K. Factors associated with HIV/AIDS diagnostic disclosure to HIV infected children receiving HAART: a multi-center study in Addis Ababa, Ethiopia. PLoS ONE. (2011) 6:1–6. 10.1371/journal.pone.0017572 21445289 PMC3061859

[38] Tucho WA Tekelehaimanot AN Habte MB. Disclosure status and associated factors among children on antiretroviral therapy in Ethiopia. Pediatr Health Med Ther. (2021) 12:299–306. 10.2147/PHMT.S314259 34211313 PMC8242142

[39] Negese D Addis K Awoke A Birhanu Z Muluye D Yifru S . HIV-positive status disclosure and associated factors among children in North Gondar, Northwest Ethiopia. Isrn Aids. (2012) 2012:1–7. 10.5402/2012/485720 24052875 PMC3767337

[40] Shallo SA Tassew M. Hiv positive status disclosure and its associated factors among children on antiretroviral therapy in west shoa zone, western ethiopia, 2019: a mixed method cross-sectional study. J Multidiscip Healthc. (2020) 13:507–17. 10.2147/JMDH.S258851 32606722 PMC7305934

[41] Doat A Negarandeh R. Disclosure of HIV status to children in Sub-Saharan Africa : a systematic review. Medicina. (2019) 55:1–12. 10.3390/medicina55080433 31382540 PMC6722600

[42] Vreeman RC. Disclosure of HIV status to children in resource-limited settings : a systematic review review. J Int AIDS Soc. (2013) 16:1–14. 10.7448/IAS.16.1.18466 23714198 PMC3665848

